# Effects of Exercise Combined with Finasteride on Hormone and Ovarian Function in Polycystic Ovary Syndrome Rats

**DOI:** 10.1155/2019/8405796

**Published:** 2019-03-13

**Authors:** Chuyan Wu, Feng Jiang, Ke Wei, Feng Lin, Zhongli Jiang

**Affiliations:** ^1^Department of Rehabilitation Medicine, The First Affiliated Hospital of Nanjing Medical University, Nanjing, China; ^2^Neonatal Department, Obstetrics and Gynecology Hospital of Fudan University, Shanghai, China; ^3^Medical Service Section, The First Affiliated Hospital of Nanjing Medical University, Nanjing, China

## Abstract

Exercise can reduce androgen and insulin levels in polycystic ovary syndrome (PCOS) patients. Finasteride is also presumed to improve the developing follicle environment. Therefore, the aim of this study was to observe the effects of the combination of exercise and finasteride therapy on hormone levels and ovarian morphology in rats with polycystic ovary syndrome. Forty female rats were randomly divided into five groups (*n* = 8 each group): the PCOS sedentary group (P-Sed), PCOS exercise group (P-Ex), PCOS finasteride and sedentary group (P-FSed), and PCOS finasteride and exercise group (P-FEx), and healthy, age-matched rats were used as controls (CO). The results indicated that the levels of FINS in the P-FEx group were significantly lower than those in the P-Sed and P-FSed groups, while the ratio of fasting blood glucose (FBG)/fasting serum levels of insulin (FINS) in the P-FEx group was significantly higher than that in the P-Sed and P-FSed groups. Compared to the P-FEx group, serum levels of TT (total testosterone) in the P-Sed and P-FSed groups were significantly increased. The thickness of the follicular membrane and the number of atresia follicles in the P-FEx and CO groups were significantly lower than those in the P-Sed group, but there was no significant difference between the P-Ex and P-Sed groups. These results show that the combined usage of exercise and finasteride does not alter the effects of exercise on increasing insulin sensitivity and reducing androgen levels. There is also a synergistic effect of exercise and finasteride on the morphology of the ovary, including a reduced number of atresia follicles and thickness of the follicular membrane.

## 1. Introduction

Polycystic ovary syndrome (PCOS) is a common endocrine syndrome among women of childbearing age and seriously affects the lives of such women [1]. Hyperandrogenemia and hyperinsulinemia are two major endocrine characteristics of polycystic ovary syndrome [2]. Additionally, acne and hirsutism are very painful complaints in adolescent women with PCOS [3, 4]. Our previous studies confirmed that 5*α*-reductase activity was enhanced in women with PCOS. Increased 5*α*-reductase activity has been shown to be associated with idiopathic hirsutism, androgenic alopecia, and acne. There are many types of drugs available for the treatment of hirsutism and acne, including oral contraceptives, antiandrogens, and reductase inhibitors [5]. Reductase inhibitors are widely used in clinical applications, including the commonly used drug finasteride. Finasteride, an antiandrogen drug, is a competitive inhibitor of type 2 5*α*-reductase, which converts testosterone into the stronger dihydrotestosterone. Small doses of finasteride are often given intermittently to adolescent PCOS women. Studies have shown that it can effectively improve the hairy symptoms of PCOS women and has no effect on metabolic parameters [6].

Exercise is a first-line treatment recommended for polycystic ovary syndrome that has been widely used in patients of all ages [7–9]. Exercise can reduce the levels of androgen and insulin in PCOS and improve the ovarian endocrine environment, which plays an important role in promoting ovulation. Finasteride is also presumed to improve the developing follicle environment. It has been shown to improve the hormonal environment around the follicle and block the development of atresia follicles [10].

In the treatment of PCOS in adolescent women, sometimes, it is necessary to combine finasteride with exercise therapy. Therefore, the purpose of this study was to investigate the effects of exercise combined with finasteride on hormone levels and ovarian morphology in rats with polycystic ovary syndrome.

## 2. Methods

The experimental protocols were approved by the Institutional Animal Care and Use Committees of Nanjing Medical University (permit number: NJMU/IA-CUC_20120210_01) and strictly followed. Forty female Wistar rats (50-70 g, 21 days old) were obtained (Beijing Vital River Laboratory Animal Technology Co., Beijing, China) and cared for according to the Guiding Principles for the Care and Use of Laboratory Animals (National Research Council of People's Republic of China, 2010). The rats were housed individually in an animal facility under controlled conditions (12 : 12 h light : dark cycle, with the light period from 8:00 am to 8:00 pm) and were given access to water. The PCOS rat model was generated by injecting testosterone propionate for 28 days and feeding a high-fat diet according to our previous studies [2]. After 28 days, the PCOS rats were randomly divided into four groups (*n* = 8 per group): a sedentary group (P-Sed), an exercise group (P-Ex), a finasteride and exercise group (P-Fex), and a finasteride and sedentary group (P-Fsed). Additionally, healthy age-matched rats (*n* = 8) were used as controls (CO). Finasteride (5 mg/kg/day) was administered by gavage in the P-Fsed and P-Fex groups once daily [11]. All the rats were sacrificed at the end of exercise between 8:00 am and 11:00 am under 12-h fasting conditions with 3% pentobarbital abdominal anesthesia followed by cervical dislocation. Ovary samples and blood were collected from all the animals.

### 2.1. Exercise Protocol

The exercised rats were forced to swim with no load for 120 min/day, 6 d/week for two weeks in a barrel filled with water at a depth of 40-50 cm maintained at 33–35°C [2, 7]. Each rat was given enough area to swim freely. The nonexercise rats' cages were kept near the barrels to equalize the effects of exercise noise.

### 2.2. Measurements of Body Weight and Body Length

The body weight and length of rats were measured weekly from 21 days of age. The body length of rats was defined as the distance from the nose to the anus. The Lee obesity index was used to reflect body fat as a parameter [LI = body weight (g) ^1/3^ × 1000/body length (cm)] [2, 12]. The bilateral ovaries of each rat were weighed, and the mean values were regarded as the ovary weight. The ovarian organ index is calculated as ovary weight/bodyweight × 10^−3^ [2, 7].

### 2.3. Serum Analysis

Following intervention, all PCOS groups (*n* = 32) and the control group (*n* = 8) were fasted for 12 h after 8:00 pm. The blood was taken from the canthus vein and used for a fasting insulin determination. The blood was taken from the tip of the tail, and blood glucose was measured by fast test paper using the Surestep Plus glucose meter (Johnson, United States). After blood glucose was measured, 3% pentobarbital was injected into the abdominal cavity, and 1-5 ml of blood from the abdominal aorta was collected and used to determine hormone levels. The blood samples obtained from rats fasting for 12 h were centrifuged at 2500 g for 10 min and stored at −80°C. Fasting serum levels of insulin (FINS), total testosterone (TT), sex hormone-binding globulin (SHBG), and dihydrotestosterone (DHT) were determined using commercial ELISA kits (Jiancheng Bioengineering Institute, Nanjing, P.R. China). The free androgen index (FAI) was calculated as follows: FAI = (TT × 100)/SHBG [13].

### 2.4. Ovarian Morphological Analysis

After separation of the bilateral ovaries, one side of the ovary was quickly put into liquid nitrogen and then stored at −80°C. After weighing, the other side of the ovary was fixed in neutral formalin solution for 24 h and then embedded in paraffin. One ovary of each rat was sectioned at the center along the long axis of the ovary and serially sectioned at 4 *μ*m from the center. We chose three serial sections with the largest areas for counting the numbers of preantral, antral, and atretic follicles and corpora lutea [7, 14]. All the sections were photographed with a camera (Leica D4000, Germany). The area of the largest section (at ×40 magnification), the number of corpora lutea (at ×100 magnification), and the thickness of the theca cell layer and granulosa cell layer (the average from 3 visual fields of follicles at ×200 magnification) were measured with Image-Pro Plus 6.0.

## 3. Statistical Analysis

SPSS statistical software (version 16.0, SPSS, Chicago, IL, USA) was used to analyze the data, and the data were described as the mean ± SD. All the data were analyzed by one-way ANOVA, and *P* < 0.05 was considered significantly different. When the ANOVA revealed significant differences among the three experimental groups, a post hoc analysis was performed by using Bonferroni's method of multiple comparisons (10 repeated tests; *P* < 0.005 was considered significant for each test).

## 4. Results

### 4.1. The Effect of Exercise Combined with Finasteride on Body Weight, Body Length, Body Mass Index, and Ovarian Organ Index in the PCOS Rats

Body weight was significantly higher in the P-FEx than in the P-Sed group, and body length was significantly greater in the P-Ex, P-FEx, and CO groups than in the P-Sed group. However, the Lee index in the P-Ex and P-FEx groups was significantly lower than that in the P-Sed group, and it was significantly higher in the P-Sed and P-FSed groups than in the CO group. The ovary organ indexes in the P-Sed and P-FSed groups were both significantly lower than in the CO group (Table 1).

### 4.2. The Effects of Exercise Combined with Finasteride on Serum Insulin and Androgens

There were no significant differences in the levels of FBG among these groups. The levels of FINS in the P-Sed and P-FSed were significantly higher than those in the other three groups, while the ratios of FBG/FINS in the P-Sed and P-FSed were significantly lower than the other three groups. In comparison with the P-Ex, P-FEx, and CO groups, the serum levels of TT in the P-Sed and P-FSed groups were significantly greater. The serum levels of SHBG in the P-Sed group were significantly lower compared with those of the other groups. The levels of FAI in the P-Sed and P-FSed groups were significantly higher than those in the P-EX and CO groups (Table 2). There was no difference in the serum DHT among these groups.

### 4.3. Observations of Ovary Slices under the Microscope

**Figure 1:**
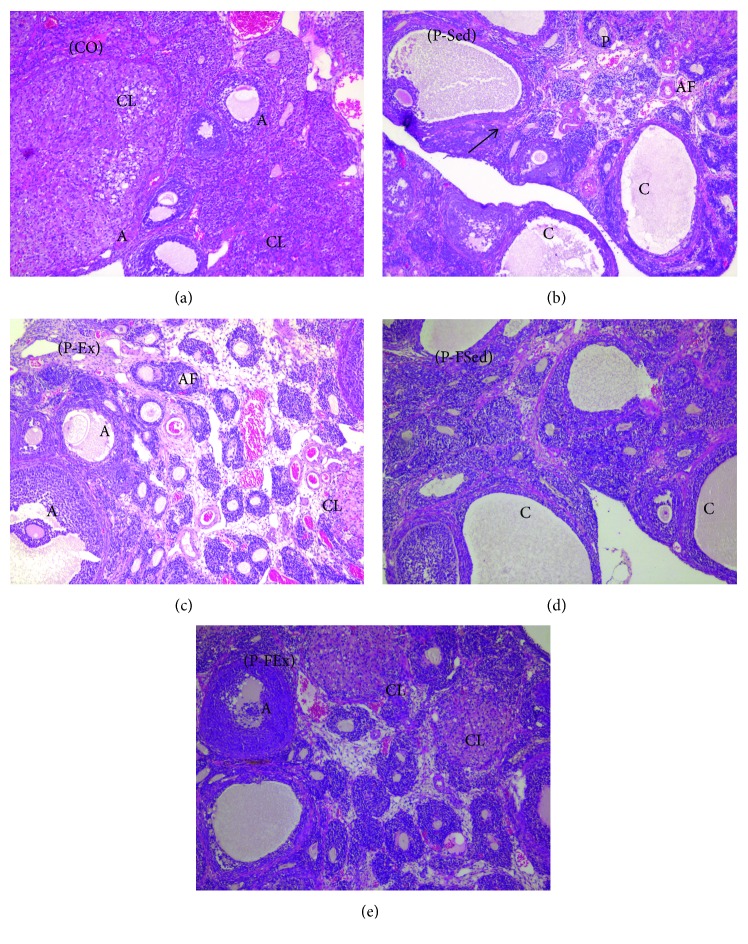
Ovarian morphology of rats (×100). (a) Control. Various stages of follicles are seen. (b) PCOS. Many preantral follicles (P) and atretic follicles (AF) with enlargement of stroma (arrow) and cystic follicles (C) are seen in the section. (c) PCOS plus exercise. Many antral follicles (A), several atretic follicles (AF), and several corpora lutea (CL) can be seen in the section. (d) PCOS plus finasteride. Different stages of antral follicles and several typical cystic structures are seen in the section. (e) PCOS plus exercise and finasteride. Several corpora lutea (CL) can be seen in the section.

In the CO, antral follicles at different stages and a large number of corpora lutea were observed. The antral follicles at different stages were observed in the P-Ex and P-FEx groups, and a small amount of luteal formation was found in the P-Ex group. The P-Sed group showed typical cystic follicular structure and many preantral and atresia follicles, while the P-FSed group showed different stages of antral follicle structure and several typical cystic structures (Figure 1).

### 4.4. Morphological Analysis of the Ovary in Each Group

Compared with the CO and P-Ex groups, the ovarian and luteal areas in the P-Sed and P-FSed groups were significantly smaller. The luteal area of the P-FEx group was also significantly smaller compared to the P-FSed and P-Sed groups. The thickness of the granular layer and the number of atretic follicles in the CO and P-FEx groups were significantly lower than those in the P-Sed group, and the numbers of antral follicles in the P-Sed and P-FSed groups were significantly lower than those in the CO group. The number of corpora lutea, the ratio of the corpus luteum area to ovarian area, the ratio of the number of corpora lutea to the total number of follicles, and the ratio of the corpus luteum area to ovarian area in the P-Sed and P-FSed groups were significantly lower than those in the CO and P-Ex groups, while the ratio of the number of atretic follicles to the total number of follicles was increased significantly in the P-FSed group compared to the CO, P-Ex, and P-FEx groups. The ratio of the corpus luteum area to ovarian area and the ratio of the number of atretic follicles in the P-FEx group were also significantly higher than those in the P-Sed group (Table 3).

## 5. Discussion

PCOS is an endocrine syndrome with high clinical heterogeneity [15–17]. This syndrome often begins during puberty [18]. Symptoms of the disease in adolescence include irregular menstruation, hirsutism, and acne [19]. These clinical manifestations greatly affect the body and mind of adolescent females, which causes a psychological burden of self-abasement and forms a vicious cycle further aggravating the disease. Both exercise and antiandrogen therapies are common methods used to treat polycystic ovary syndrome [16, 20–22]. However, it is not clear how the combination of these two factors affects the female hormone levels and the ovary. The PCOS rat model is characterized by hyperinsulinemia and hyperandrogenemia [2, 23]. The morphology of the ovaries includes the morphology of a polycystic follicle, which consists of a thin layer of follicle granules, a thickening of the follicular membrane, and increasing follicular follicles [2].

Numerous animal experiments and human studies have confirmed that exercise treatment can reduce weight [21] and improve hyperinsulinemia and high androgen levels [22] in individuals exhibiting PCOS. In addition, the ovaries can be restored to promote ovulation by exercise. Among the antiandrogen drugs, finasteride, a type 2 reductase inhibitor, has been shown by many studies to exhibit a higher than average safety level [19, 24–26]. Compared with the type 1 reductase inhibitor dutasteride, previous experiments with finasteride have proved that there is no significant difference in clinical efficacy and related parameters [27, 28]. However, recent studies have shown that taking dutasteride can increase body fat and decrease insulin sensitivity, whereas finasteride has no such effect and can improve insulin resistance [29, 30]. In the present study, the finasteride group showed no significant difference in body mass index; however, it showed an increase in body weight compared with the nonfinasteride group.

Finasteride alone cannot improve insulin resistance in PCOS rats. Fortunately, it also does not aggravate insulin resistance and it did not affect the action of exercise to improve insulin resistance in the P-FEx group. Finasteride inhibited testosterone conversion to dihydrotestosterone, resulting in an increase in total testosterone in the two finasteride groups. In addition, a large number of population experiments have confirmed that sex hormone-binding protein was closely related to impaired glucose tolerance [31, 32]. In this study, it was also found that the levels of sex hormone-binding protein in the two exercise groups were significantly higher than those in the two sedentary groups. The current study also confirmed that finasteride has no significant effect on improving insulin resistance.

In a population study in 2004, 31 PCOS women were given oral finasteride or the same dose of placebo. The experiment concluded that the PCOS women who took finasteride did not show a significant difference in ovarian capacity compared with the control group [33]. In our study, the PCOS rats in the P-FEx group showed a significant reduction in the area of the corpus luteum. Although the area of the ovary was decreased, there was no significant difference compared with the P-Ex group. The growth of follicles is affected by aromatase and reductase and is particularly affected by their metabolites, estradiol and dihydrotestosterone. Follicular growth is the result of interactions between endocrine, autocrine, and paracrine factors. High levels of DHT in the follicle block the cell signaling pathway. Studies have shown that androgen receptor blockers and testosterone antibodies inhibit follicular atresia [10, 34]. Our experimental results showed that the P-FEx rats compared with the P-Sed rats primarily displayed decreased atresia follicles and decreased follicle film thickness, which is consistent with the inference that finasteride can reduce follicle atresia. However, the improvement of ovarian morphology in these two aspects was not obvious in the P-Ex group. A possible mechanism is that the growth and development of follicles can be affected by competition between aromatase and 5*α*-reductase.

In conclusion, this study investigated the obesity PCOS rat model induced by testosterone propionate combined with a high-fat diet. Exercise plus finasteride or either intervention alone was used to observe the effects of exercise, finasteride, and their combination on serology and ovarian morphology in the PCOS rats. The results showed that the combined use of finasteride and exercise did not alter the effect of exercise on increasing insulin sensitivity and reducing androgen levels. Furthermore, there is a synergistic effect of finasteride and exercise on the morphology of the ovary as the number of atresia follicles, and the thickness of the follicular membrane can be reduced. However, due to the small number of animals tested and the difference with the model of a chronic hyperandrogenism as in PCOS women, the effect of finasteride on the PCOS patient population needs further study.

## Figures and Tables

**Table 1:** Comparisons of weight, length, Lee index, and ovary organ index.

	CO	P-Sed	P-Ex	P-FSed	P-FEx
Body weight (g)	199.17 ± 15.40	185.00 ± 5.83	190.71 ± 6.73	202.83 ± 13.36	205.17 ± 9.04^b^
Body length (cm)	20.88 ± 0.99	19.31 ± 0.72^a^	20.47 ± 0.64^b^	20.08 ± 0.56	20.87 ± 0.27^b^
Lee index	280.07 ± 16.14	295.27 ± 9.30^a^	281.39 ± 9.83^b^	292.53 ± 6.62^a^	282.62 ± 4.23^b^
Ovary organ index (×10^−3^)	0.35 ± 0.04	0.24 ± 0.05^a^	0.30 ± 0.03	0.24 ± 0.02^a^	0.30 ± 0.02

Values are the means ± SD. ^a^Compared with the CO group, *P* < 0.005. ^b^Compared with the P-Sed group, *P* < 0.005.

**Table 2:** Levels of insulin and androgen in each group.

	CO	P-Sed	P-Ex	P-FSed	P-FEx
FBG (mmol/l)	3.62 ± 0.99	3.41 ± 0.48	3.47 ± 0.30	3.05 ± 0.49	3.33 ± 0.53
FINS (*μ*IU/ml)	11.10 ± 0.79	12.76 ± 1.10^a^	11.10 ± 0.87^b^	12.42 ± 1.40^a,c^	11.04 ± 1.11^b,d^
FBG/FINS	0.33 ± 0.09	0.26 ± 0.04^a^	0.32 ± 0.05^b^	0.24 ± 0.02^a,c^	0.31 ± 0.07^b,d^
TT (nmol/l)	34.26 ± 2.11	38.52 ± 3.64^a^	34.56 ± 5.37^b^	38.39 ± 1.13^a,c^	36.66 ± 1.42^b,d^
DHT (nmol/l)	30.18 ± 2.39	30.29 ± 3.01	31.62 ± 2.28	30.03 ± 4.12	30.25 ± 2.59
SHBG (nmol/l)	52.78 ± 2.71	44.76 ± 1.08^a^	51.85 ± 5.22^b^	48.53 ± 4.22	50.03 ± 2.74^b^
FAI (nmol/l)	68.06 ± 3.81	85.95 ± 7.18^a^	66.96 ± 10.09^b^	79.26 ± 7.04^a,c^	73.24 ± 6.78

Values are the means ± SD. ^a^Compared with the CO group, *P* < 0.005. ^b^Compared with the P-Sed group, *P* < 0.005. ^c^Compared with the P-Ex group, *P* < 0.005. ^d^Compared with the P-FSed group, *P* < 0.005.

**Table 3:** Analysis of morphological parameters of the five groups of rat ovaries.

	CO	P-Sed	P-Ex	P-FSed	P-FEx
Ovary area	3.58 ± 0.54	2.36 ± 0.84^a^	3.50 ± 0.67^b^	2.15 ± 0.54^a,c^	2.95 ± 0.29
Corpus luteum area (mm^2^)	0.64 ± 0.09	0.18 ± 0.07^a^	0.61 ± 0.10^b^	0.19 ± 0.07^a,c^	0.58 ± 0.06^b,d^
Thickness of granular layer	39.14 ± 5.89	17.06 ± 1.61^a^	47.66 ± 5.96^b^	15.51 ± 1.25 ^a,c^	41.28 ± 9.09^b,d^
Thickness of theca layer	12.85 ± 3.83	19.17 ± 2.82^a^	15.36 ± 2.18	17.32 ± 4.17	13.36 ± 2.18^b^
Number of preantral follicles	3.80 ± 0.84	4.80 ± 1.48	3.60 ± 1.52	5.25 ± 1.26	3.33 ± 0.58
Number of antral follicles	6.60 ± 1.14	4.80 ± 0.84^a^	6.20 ± 0.84	4.25 ± 1.26^a^	5.67 ± 1.15
Number of atretic follicles	4.20 ± 1.79	8.20 ± 1.30^a^	5.20 ± 0.45	7.05 ± 4.79	4.00 ± 1.00^b^
Total number of follicles	14.60 ± 3.13	17.80 ± 1.64	15.00 ± 1.58	15.75 ± 3.86	15.00 ± 1.00
Number of corpora lutea	3.49 ± 0.50	1.33 ± 0.58^a^	3.20 ± 0.84^b^	1.75 ± 0.50^a,c^	2.67 ± 0.58
Ratio of the corpus luteum area to the ovarian area	0.18 ± 0.04	0.08 ± 0.01^a^	0.18 ± 0.04^b^	0.09 ± 0.02^a,c^	0.15 ± 0.03^b,d^
Ratio of the number of corpora lutea to the total number of follicles	0.24 ± 0.09	0.05 ± 0.01^a^	0.21 ± 0.06^b^	0.10 ± 0.04^a,c^	0.18 ± 0.05^b^
Ratio of the number of atretic follicles to the total number of follicles	0.29 ± 0.07	0.46 ± 0.05^a^	0.35 ± 0.03	0.47 ± 0.16^a,c^	0.26 ± 0.06^b,d^

Values are the means ± SD. ^a^Compared with the CO group, *P* < 0.005. ^b^Compared with the P-Sed group, *P* < 0.005. ^c^Compared with the P-Ex group, *P* < 0.005. ^d^Compared with the P-FSed group, *P* < 0.005.

## Data Availability

The data used to support the findings of this study are available from the corresponding author upon request.

## Abbreviations

PCOS: Polycystic ovary syndrome
FBG: Fasting blood glucose
FINS: Fasting serum levels of insulin
TT: Total testosterone
SHBG: Sex hormone-binding globulin
DHT: Dihydrotestosterone
FAI: Free androgen index.
